# Structure-Gas Barrier Property Relationship in a Novel Polyimide Containing Naphthalene and Amide Groups: Evaluation by Experiments and Simulations

**DOI:** 10.3390/ma14061402

**Published:** 2021-03-13

**Authors:** Yi Zeng, Yiwu Liu, Jinghua Tan, Jie Huang, Junjie Liu, Ao Tang, Chengliang Chen, Hong Chen

**Affiliations:** 1 State Key Laboratory for Powder Metallurgy, Centre South University, Changsha 410083, China; zengyi2426@163.com; 2 National and Local Joint Engineering Center of Advanced Packaging Materials R & D Technology, Key Laboratory of Advanced Packaging Materials and Technology of Hunan Province, School of Packaging and Materials Engineering, Hunan University of Technology, Zhuzhou 412007, China; tjh@hut.edu.cn (J.T.); huangjie3@sjtu.edu.cn (J.H.); junjie0108@whu.edu.cn (J.L.); tangao1234@163.com (A.T.); chenchengliangc@163.com (C.C.)

**Keywords:** polyimide, naphthalene ring, amide group, barrier properties, molecular simulation

## Abstract

In order to meet the increasingly stringent requirements for heat resistance and barrier properties in the packaging and electronic device encapsulation field. A high-barrier polyimide (NAPPI) contains naphthalene ring and amide group was prepared by polymerization of a novel diamine (NAPDA) and pyromellitic dianhydride. The structure and properties of diamine monomers and polymers were characterized. Results show that the NAPPI exhibits superior barrier properties with extremely low water vapor and oxygen transmission rate values of 0.14 g·m^−2^·day^−1^ and 0.04 cm^3^·m^−2^·day^−1^, respectively. In addition, the NAPPI presents outstanding mechanical properties and thermal stability as well. This article attempts to explore the relationship between NAPPI structure and barrier properties by combining experiment and simulation. Studies on positron annihilation lifetime spectroscopy, Wide angle X-ray diffractograms and molecular dynamics simulations prove that the NAPPI has smaller interplanar spacing and higher chain regularity. In addition, the strong chain rigidity and interchain cohesion of NAPPI due to the presence of the rigid naphthalene ring and a large number of hydrogen bond interactions formed by amide groups result in compact chain packing and smaller free volume, which reduces the solubility and diffusibility of small molecules in the matrix. In general, the simulation results are consistent with the experimental results, which are important for understanding the barrier mechanism of NAPPI.

## 1. Introduction

Polymers which provide barriers toward gases play a key role in packaging application and electronic encapsulation due to their lightweight, low cost, flexibility, and easily processing features [1,2,3,4,5]. However, compared with traditional barrier materials such as glass and metals or ceramics, common polymer barrier materials have relatively high permeability and comparatively low thermal resistance, this limits their application in the encapsulation of novel electronic devices such as flexible organic light emitting diodes (OLEDs) and flexible solar cells [6,7,8,9,10]. Polyimide, as a new type of polymer with unparalleled comprehensive properties, exhibits excellent heat resistance has received extensive attention. More importantly, the chemical structure of polyimide can be designed at the molecular level [11,12,13,14,15]. Studies have shown that the significant improvement in barrier performance may be caused by the subtle structural changes in the polymer backbone [16,17,18]. This provides the possibility to synthesize a novel polymer material with high heat resistance and high barrier properties.

The rigidity of polymer chains and chain packing can be considered as the key factors that have a direct impact on barrier properties; the more efficient chain packing may reduce the free volume, if the free volume size smaller than penetrant and the rigidity of the chain is strong enough, the penetrant molecules will be difficult to diffusion [19,20,21,22,23]. Brennan et al. [24] found that the aromatic unit in the polymer backbone becomes less bulky and more planar or compact; the polymer has better gas barrier properties. For example, the introduction of high planarity of naphthalene may result in more efficient chain packing, thus reducing free volume; in addition, naphthalene ring can improve the rigidity of molecular chain, and reduce the permeability [24,25]. This is the same as the case of polyethylene naphthalate (PEN) and polyethylene terephthalate (PET). The combination of the two polymers is similar. The difference is that the benzene ring in PET is replaced by naphthalene ring with a larger rigid planar structure, so the PEN has better barrier performance [26,27,28]. Brennan et al. [29] also pointed out that amide unit has a great influence on the improvement of high barrier properties of polymers. The existence of amide group can increase the hydrogen bond interaction in polymer matrix. Hydrogen bond will bring strong intermolecular cohesion, which makes the optimal chain packing, thus improving the barrier properties [29,30,31].

**Figure 1:**
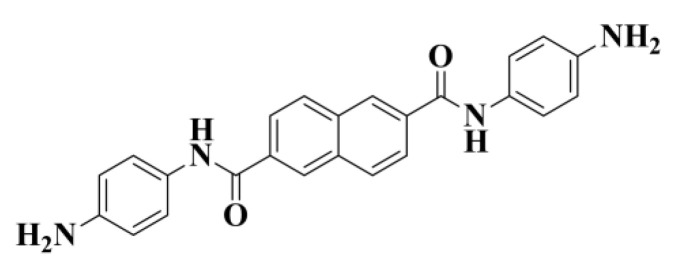
Structural formula of NAPDA.

Referring to the relevant literature and based on the previous experience, this study aimed at a kind of polyimide which containing amide groups and naphthalene rings as the object. A diamine monomer (NAPDA) containing naphthalene group and amide group was synthesized through amidation reaction and reduction reaction. Then, polyimide (NAPPI) obtained by polymerization of NAPDA and pyromellitic dianhydride. Figure 1 shows the structural formula of the novel diamine NAPDA.

To further improve the barrier properties and develop high barrier polymer films, it is necessary to have a fundamental understanding of the barrier mechanism [32]. However, to clarify the polymer structure and barrier properties at an atomistic/molecular level is experimentally intractable. Fortunately, it has become more convenient to use molecular simulation method to study the relationship between microstructure and macroscopic properties due to the upgrading computational resources. This provides a unique opportunity to study the barrier mechanism of polymers at the molecular level [33,34,35,36]. The parameters related to the barrier properties, such as the size and distribution of free volume, penetrant trajectories, diffusion coefficient, and solubility coefficient can be obtained by molecular simulation [37,38,39,40].

In this study, we performed to discuss the fundamental structure-property relationships of the NAPPI film. Wide angle X-ray diffractograms (WAXD) were used to discuss the crystallinity of polyimide films, and then combined with molecular simulation and positron annihilation lifetime spectroscopy (PALS) to evaluate the aggregate structure of polymers. Molecular simulations were performed to further estimate the free volume distribution, glass transition temperature, cohesive energy density, and hydrogen-bonding interaction. The mean square displacement (MSD) and diffusion trajectory of gas molecules were researched by molecular dynamics (MD), and the sorption isotherm was studied by Grand Canonical Monte Carlo (GCMC) simulations. The purpose is to clarify the mechanical insights about the excellent barrier properties of the novel polyimide and ravel the essential governing factors, which will be helpful to the rational design of novel high-barrier films.

## 2. Materials and Methods

### 2.1. Materials

2, 6-naphthalenedicarboxylic acid (purity: 99%, J&K Scientific Ltd., Beijing, China), 4-nitroaniline (purity: 99%, Aladdin Reagent Co., Ltd., Shanghai, China), 10% palladium on carbon (10 wt% Pd/C, Alfa Aesar Chemical Co., Ltd., Shanghai, China), triphenyl phosphate (TPP, purity: 98%, Macklin Biochemical Co., Ltd., Shanghai, China), argon (purity: 99.999%, Hualong Special Gas Co., Ltd., Zhuzhou, Hunan, China), nitrogen (purity: 99.999%, Hualong Special Gas Co., Ltd., Zhuzhou, Hunan, China) and hydrazine monohydrate (NH_2_NH_2_·H_2_O, purity: 95%, J&K Scientific Ltd., Beijing, China) were used directly without further treatment. Pyromellitic dianhydride (PMDA, Alfa Aesar Chemical Co., Ltd., Shanghai, China) and 4, 4′-diaminodiphenyl ether (ODA, Alfa Aesar Chemical Co., Ltd., Shanghai, China) was heated at 125 °C in vacuum for 4 h prior to use. N-Methyl pyrrolidone (NMP), dimethylformamide (DMF), methanol, ethanol and pyridine (Py) were purchased from Guangzhou Chemical Reagent Factory (Guangzhou, Guangdong, China), before use, all solvents was dehydrated with molecular sieves.

### 2.2. Synthesis of Monomers and Polymers

#### 2.2.1. Synthesis of N^2^, N^6^-bis(4-nitrophenyl)naphthalene-2,6-dicarboxamide (NAPDN)

Dissolve 3.459 g (0.016 mol) of 2, 6-naphthalenedicarboxylic acid and 11.051 g (0.08 mol) of 4-nitroaniline in 100 mL NMP/Py (4:1) mixed solution, then 9.936 g (0.032 mol) TPP were added under argon protection. The mixture was precipitated by addition of 400 mL methanol after heated to 120 °C refluxed for 6 h. The precipitate was filtered and washed thoroughly with methanol. NAPDN was obtained as a yellow powder through purification by recrystallization in DMF, the yield of the NAPDN is about 80%. IR (KBr): 754–1110 cm^−1^ (Ar–H stretching), 1329, 1545 cm^−1^ (–NO_2_ stretching). ^1^H NMR (400 MHz, DMSO-*d*_6_, δ): 10.64 (s, 2H), 8.65 (s, 2H), 8.32–8.18 (m, 2H), 8.10 (d, *J* = 8.6 Hz, 2H), 7.84 (d, *J* = 8.4 Hz, 4H), 7.70–7.49 (m, 4H). MS (EI, m/z): 456 ([M]^+^, calcd for C_24_H_16_N_4_O_6_, 456.11).

#### 2.2.2. Synthesis of N^2^, N^6^-bis(4-aminophenyl)naphthalene-2,6-dicarboxamide (NAPDA)

4.564 g (0.01 mol) of NAPDN and 300 mL of ethanol was added to a three-necked flask (Beijing Synthware glass Co., Ltd., Beijing, China) under argon atmosphere. When the temperature rises to 80 °C, add a catalytic amount of Pd/C, and then slowly add 8 mL of hydrazine hydrate dropwise. After refluxing for 24 h, the reaction mixture was filtered. The filter cake was dissolved in DMF and then filtered again to remove the Pd/C catalyst. The filtrate is poured into water; a large quantity of precipitation will precipitate out, and filter to obtain a crude product after standing still. The collected solid was heated at 80 °C for 8 h in vacuum. The yield is about 87%. The FTIR, NMR and MS characteristic spectrum data of the product are as follows: IR (KBr): 816–1171 cm^−1^ (Ar–H stretching), 1258 cm^−1^ (C–N stretching), 1610 cm^−1^ (δ N–H), 1641 cm^−1^ (C=O stretching), 3370 cm^−1^ (N–H stretching). ^1^H NMR (400 MHz, DMSO-d_6_, δ): 10.13 (s, 2H, –NH), 8.59 (s, 2H, Ar H), 8.23–8.13 (m, 2H, Ar H), 8.08 (d, *J* = 8.4 Hz, 2H, Ar H), 7.46 (d, *J* = 8.4 Hz, 4H, Ar H), 6.59 (d, *J* = 8.4 Hz, 4H, Ar H), 4.97 (s, 4H, -NH_2_); ^13^C NMR (100 MHz, DMSO-d_6_, δ): 164.53 (C7), 145.32 (C11), 134.14 (C3), 133.25 (C1 and C6), 128.96 (C5), 128.14 (C8), 127.31 (C2), 125.22 (C4), 122.20 (C9), 113.72 (C10); MS (EI, m/z): 396 ([M]^+^, calcd for C_24_H_20_N_4_O_2_, 396.16).

#### 2.2.3. Synthesis of Polyimide (NAPPI)

First, under the protection of argon, dissolve 0.7929 g (2 mmol) of NAPDA and 0.4362 g (2 mmol) of PMDA in 14 mL of DMF, and the mixture was continuously stirred for 6 h in an ice bath to prepare poly(amic acid) (PAA) solution with 8 wt% solid content. The homogeneous and transparent PAA solution is then uniformly coating onto a clean glass sheet after defoaming treatment. The PAA-coated glass sheet was immediately placed in a vacuum drying oven (Shanghai Experimental Instrument Factory Co., Ltd., Shanghai, China) for thermal imidization. After heating from room temperature to 400 °C, and holding at 100, 200, 300, and 400 °C for 30 min during the period, respectively, the polyimide film was finally obtained.

### 2.3. Measurements

Mass spectra (MS) were generated by Thermo DSQ II mass spectrometer (Thermo Fisher Scientific, Waltham, MA, USA). Nuclear magnetic resonance spectroscopy (NMR) was performed on Bruker Avance AV-400 spectrometer (Bruker Corporation, Fallanden, Switzerland) at 400 and 101 MHz, respectively. Nicolet iS10 Fourier Transform Infrared (FTIR) spectrometer (Thermo Fisher Scientific, Waltham, MA, USA) was used to obtain the infrared spectra of monomers and films. Using Rigaku, Ultema III X-ray diffractometer (Rigaku Corporation, Tokyo, Japan) obtained the WAXD spectrum under CuKα radiation. Density was obtained with a MIRAGE SD-200L ALFA electronic density balance (Alfa Mirage Corporation, Osaka, Japan).

Thermogravimetric analyses (TGA) were implemented on a TA TGA55 thermal analyzer (TA instruments, New Castle, DE, USA), the sample was heated to 800 °C under the conditions of a nitrogen flow rate of 40 mL/min and a heating rate of 20 °C/min. Dynamic mechanical analysis (DMA) was used to evaluate the thermomechanical properties of polyimide with a TA DMA850 dynamic mechanical analyzer (TA instruments, New Castle, DE, USA) in tensile mode at a frequency of 1 Hz, and the temperature raised to 550 °C at a rate of 5 °C/min. Coefficient of thermal expansion (CTE) was measured by TA TMA Q400 Thermalmechanical analyzer (TA instruments, New Castle, DE, USA) from 20 to 350 °C at a step of 5 °C/min. CMT6103 electronic universal testing machine (New Sansi Material Testing Co., Ltd. Shenzhen, China) were used to test the mechanical properties of polyimide followed GB/T16421-1996, the specimen size was 10 mm × 100 mm with a test speed of 2 mm/min.

Oxygen transmission rate (OTR) was investigated by the Mocon OX-TRAN 2/21 devices ((Mocon Corporation, Minneapolis, MN, USA) following the ASTM-D3985 standard method, the test is performed at 23 °C with a relative humidity (RH) of 0% [41]. Water vapor transmission rate (WVTR) was analyzed using Mocon PERMATRAN-W^®^ 3/33 devices ((Mocon Corporation, Minneapolis, MN, USA) followed ASTM F-1249, the test is performed at 37.8 °C with 90% RH [42]. To reduce deviation, the films were covered by aluminum foil mask ((Mocon Corporation, Minneapolis, MN, USA) with an area 5 cm^2^ central opening on both sides to make a sandwich-like sample.

The positron lifetime measurement of the sample of a multilayer film stack with a size of 10 mm × 10 mm × 1.5 mm was performed by PALS to calculate the free volume of polyimide. For details, please refer to our previous work [31].

### 2.4. Molecular Simulation

**Figure 2:**
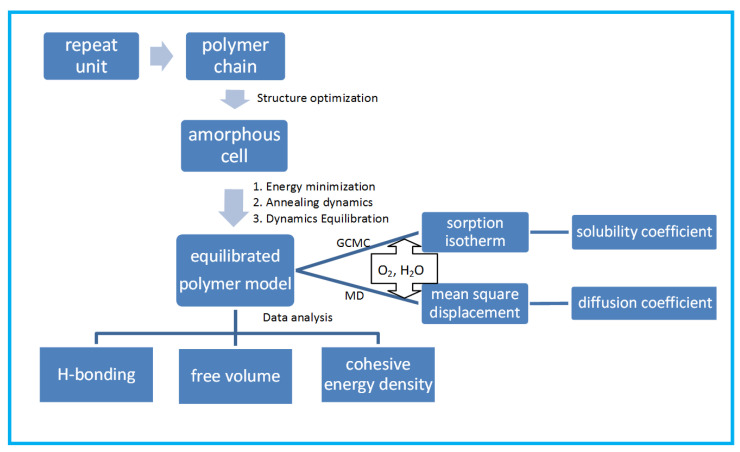
Molecular simulation process of polyimide (GCMC stands for Grand Canonical Monte Carlo Method, MD stands for Molecular Dynamics Simulation).

A molecular simulation study of polyimide was carried out based on the COMPASS forcefield by using Material Studio 8.0 software (Accelrys Software Inc., San Diego, CA, USA). The simulation process is shown in Figure 2. First, a polyimide chain with 25 repeating units was constructed using the “Build” function followed by structure optimization. Then, the amorphous cell of polymer with periodic boundary conditions are established, which is composed of 5 polymer chains. Next, the energy minimization, annealing dynamics and molecular dynamics equilibrium simulations were carried out on the amorphous cell in sequence. Energy minimization is achieved through the smart minimizer method, the maximum iterations is set to 10^5^. The optimized amorphous cell is relaxed by the annealing dynamics, which uses the isothermal-isobaric ensemble (NPT) to perform a loop of heating from 300 to 1000 K and then cooling to 300 K (heating or cooling in steps of 50 K), and the loop executes ten times. After annealing, the system will undergo a dynamics equilibration procedure in three stages. The system will first be heated to a fully relaxed configuration of 1000 K, and then gradually reduce to 600 K in steps of 100 K, Then use the same method to reduce to 400 K in steps of 50 K, and finally to 300 K in steps of 25 K. The dynamics simulations of 250 ps canonical ensemble (NVT) and 250 ps NPT were performed for each step of the equilibrium. After completing the above procedures, the system was further stabilized at 300 K through 500 ps NVT and 500 ps NPT to guarantee a fully equilibrium state. The equilibrium state can be judged by energy as a function of time and density as a function of time in simulation system. All simulations were performed at a pressure of 0.0001 GPa; the atom based, and Ewald summation method, were applied to calculate the van der Waals interactions (15.5 Å cutoff) and electrostatic interactions; the thermostat and the barostat are controlled by the Nosé method and the Berendsen method, respectively.

Data analysis was performed after obtaining the equilibrium conformation of the polymer. The free volume and its distribution within the polyimide film were calculated by using Connolly task. In order to explain the hydrogen bond interaction, the radial distribution functions (RDF) was analyzed. In addition, the cohesive energy density of the polyimide was also calculated.

The sorption isotherms are used to analyze the solution behavior of H_2_O and O_2_ in polyimide, which is obtained by GCMC simulation using the Sorption module. In addition, the simulation is based on the Metropolis algorithm. In this simulation, the sorption isotherms of H_2_O and O_2_ in NAPPI and Kapton (obtained by polymerization of ODA and PMDA) under a pressure range of 10 to 3000 kPa at 298 K were investigated, where the equilibration steps are set to the 1 × 10^5^, and the production steps is 1 × 10^6^.

The diffusion behavior of gas molecules in polyimide can be investigated by MD simulation. First, randomly insert 10 H_2_O and O_2_ when constructing the polymer amorphous cell, and then perform dynamics simulations of the new cell according to the same method described above (namely energy minimization, annealing dynamics, and dynamics equilibration) to make the structure reach an equilibrium state. Subsequently, the system was further equilibrium for 10,000 ps in the microcanonical ensemble (NVE) at 298 K to analyze MSD and the diffusion trajectories of H_2_O and O_2_.

## 3. Results and Discussion

### 3.1. Synthesis and Characterization of Diamines and Intermediates

**Scheme 1:**
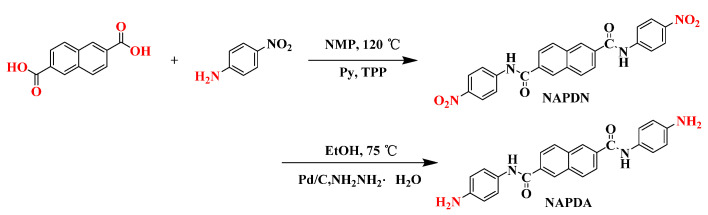
Synthesis routes of NAPDA.

**Figure 3:**
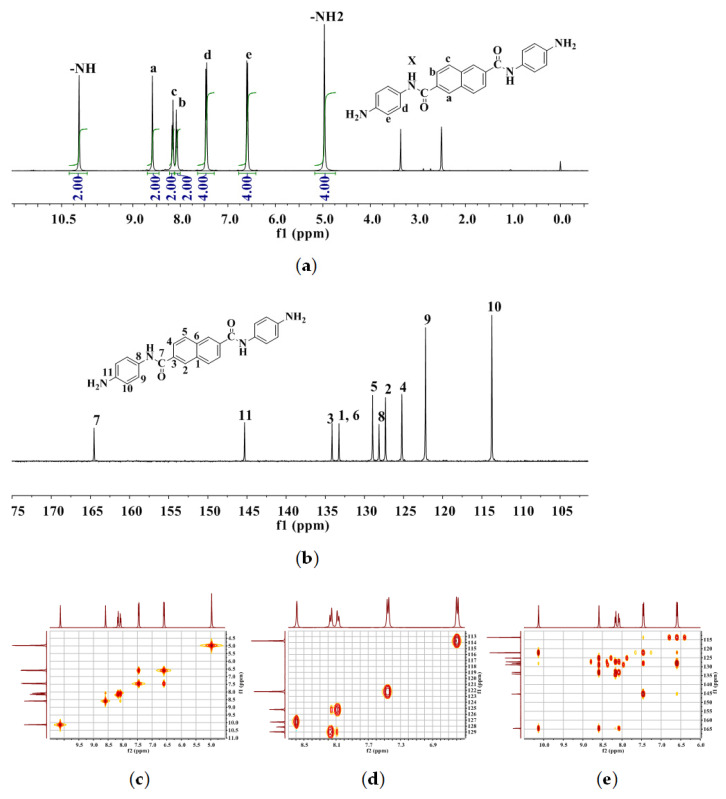
(**a**) ^1^H Chemical Shifts, f1 (ppm) in ^1^H NMR, (**b**) ^1^^3^C Chemical Shifts, f1 (ppm) in ^13^C NMR, (**c**) H–H COSY, (**d**) C–H QC and (**e**) C–H BC spectra of NAPDA.

Scheme 1 shows the synthesis process of NAPDA. First, The NAPDN moiety was synthesized by the amidation reaction of 2, 6-naphthalene dicarboxylic acid with 4-nitroaniline diamine, and then the NAPDA was synthesized by reduction reaction of NAPDN. The structures of NAPDA and NAPDN were identifying by using NMR, FTIR and MS. Figure 3 is the ^1^H NMR spectra, ^13^C NMR spectra and two-dimensional NMR spectra of NAPDA, respectively. The NMR spectra of NAPDN are shown in Figure S1. As it shown, each proton and carbon was assigned to the NMR spectra of the diamine monomer and the intermediates. The mass spectra and FTIR spectra of NAPDN and NAPDA were shown in Figures S2–S4, respectively. The spectral results are consistent with the proposed molecular structures of NAPDN and NAPDA. All these proved that the diamine monomer NAPDA has been successfully synthesized through amidation reaction and reduction reaction.

Figure S5 show the thermal properties evaluated by DSC and TGA of the synthesized NAPDA, Table S1 lists the corresponding data. The melting point (*T_m_*) of NAPDA was 333 °C measured by DSC. The TGA test found that the temperature at 5% and 10% weight loss (*T_d5%_* and *T_d10%_*) under N_2_ was 386 and 405 °C, respectively. The high melting point and high weight loss temperature indicate that the NAPDA exhibits excellent thermal stability.

### 3.2. Synthesis and Characterization of Polyimides

**Scheme 2:**
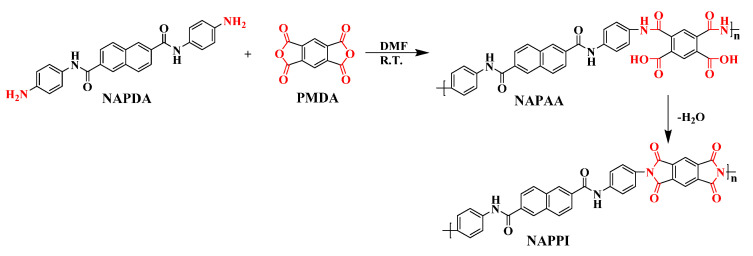
Synthesis routes of polyimides.

**Figure 4:**
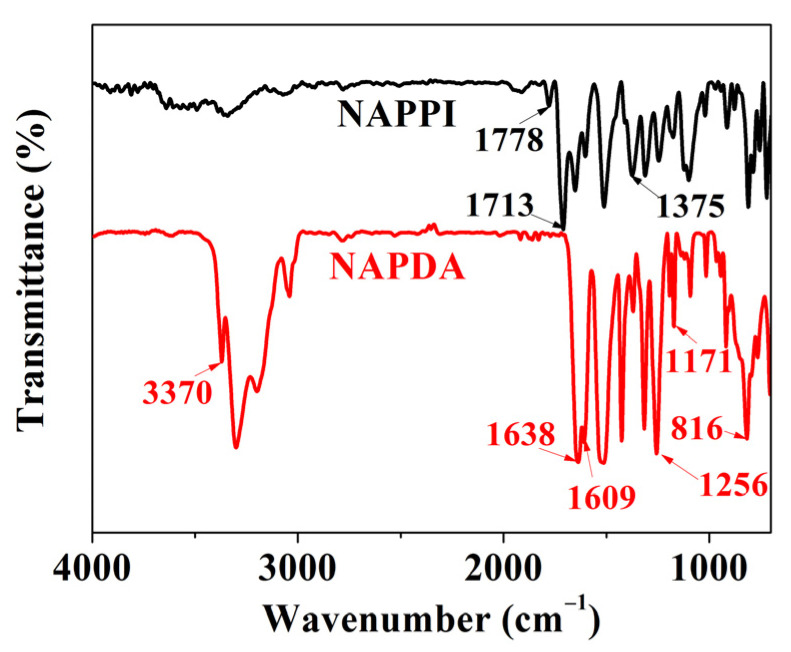
FT-IR spectrum of NAPDA and NAPPI.

NAPPI follows the synthesis steps shown in Scheme 2. First, the prepolymer NAPAA is formed by ring-opening polymerization, which is completed in DMF using equimolar equivalents of NAPDA and PMDA. Subsequently, the polyimide NAPPI is formed by thermal cyclodehydration. The transition from PAA to polyimide accomplished by continuously heating poly(amic acid)s film from 100 to 400 °C in a vacuum. The FTIR spectrum of NAPPI is shown in Figure 4. The characteristic absorption peak around 1778 and 1713 cm^−1^ are connected to asymmetric and symmetric stretching of the C=O, respectively. There is an obvious C–N bond stretching vibration characteristic absorption peak at 1375 cm^−1^, these are typical characteristic absorption peaks of polyimide. In addition, the tensile vibration absorption peak at 3370 cm^−1^ and bending vibration absorption peak at 1609 cm^−1^ of N–H bond was not found in the spectrum of NAPPI. These results indicated that the reaction of PMDA and NAPDA was successful, and the PAA was completely converted to polyimide through the imidization reaction.

**Figure 5:**
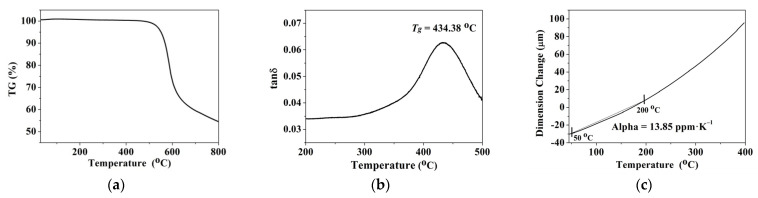
(**a**) Thermogravimetric analyses (TGA), (**b**) Dynamic mechanical analysis (DMA), and (**c**) Thermalmechanical analysis (TMA) curves of the NAPPI film.

TGA, DMA, and Thermalmechanical analysis (TMA) were used to investigate the thermal properties of NAPPI. Figure 5 shows its correlation curve (a) TGA, (b) DMA, and (c) TMA. Related data are recorded in Table 1. For comparison, the properties of Kapton film obtained by polymerization of 4, 4′-diaminodiphenyl ether (ODA) and PMDA are introduced. NAPPI loses 10% of its weight at 568 °C and only 5% at 551 °C. This indicates that NAPPI has excellent heat resistance as Kapton film. Figure 5b manifest that NAPPI has a relatively high *T_g_* close to 434 °C, which is measured by DMA. In addition, Figure 5c shows that NAPPI had a fairly low CTE of 13.85 ppm·K^−1^ between 50 and 200 °C. These results all indicate that NAPPI has higher thermal stability than commonly commercial polymers. Due to the presence of the rigid naphthalene ring, the rigidity of the polymer chain increased, and the amide group with strong polarity can further enhance the chain rigidity and form a stronger interchain cohesion. The rigid chain will resist thermally induced vibrations, which is an important reason for the excellent thermal stability of NAPPI [43]. In addition, as shown in Figure S6, NAPPI can be rolled into a barrel, which is indicating it retains good toughness. The tensile strength and modulus of NAPPI reached 138 MPa and 3.5 GPa, respectively, which proves that it has good mechanical properties. The mechanical performance curve is shown in Figure S7.

### 3.3. Barrier Properties

Table 2 lists the OTR and WVTR of the polyimide films. The OTR and WVTR values of NAPPI are as low as 0.04 cm^3^·m^−2^·day^−1^ and 0.14 g·m^−2^·day^−1^, respectively. This is nearly three to four orders of magnitude below the Kapton. There is no doubt that NAPPI has demonstrated unparalleled barrier performance. Its oxygen and water vapor permeability is much lower than common commercial high-barrier polymer films such as PET, PEN, polyvinylidene chloride (PVDC), nylon-6 (PA-6) and ethylene(vinyl alcohol) (EVOH). Although it still cannot meet the barrier performance requirements of OLEDs for substrates, it is fully capable of solar cell encapsulation, E-paper and other high-performance packaging. Due to its excellent barrier properties and thermal stability, NAPPI film is undoubtedly one of the more reliable substrate material candidates than the currently commonly used commercial high barrier polymers.

### 3.4. Crystallinity Analysis

**Figure 6:**
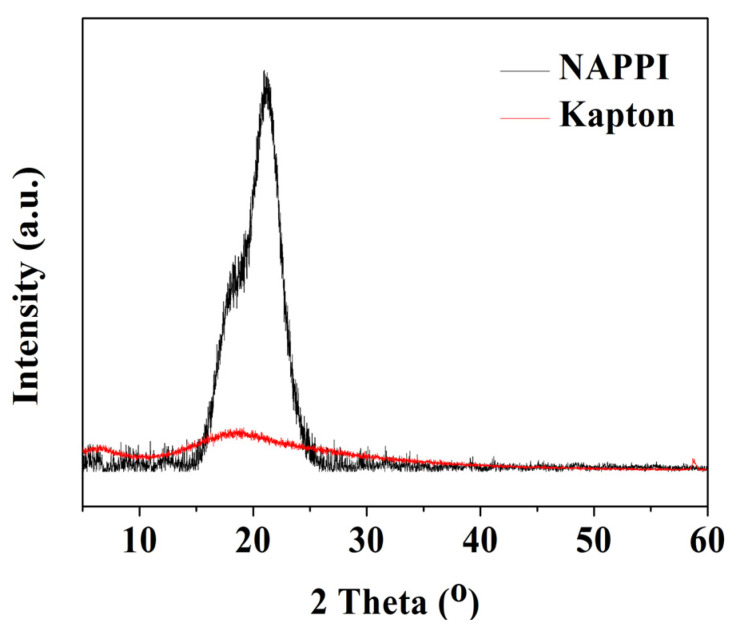
Wide angle X-ray diffractograms (WAXD) curves of the Kapton and NAPPI.

**Figure 7:**
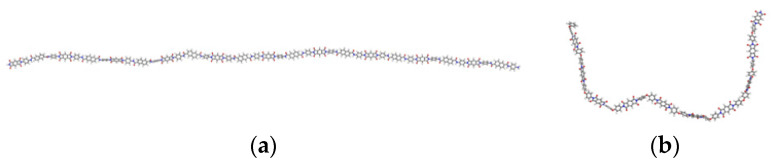
Single chain configurations (10 repeat units) of the (**a**) NAPPI and (**b**) Kapton [23].

The crystallinity and the inter-chain spacing of the NAPPI and Kapton films were examined by WAXD, and the WAXD pattern is shown in Figure 6. The diffraction peak of NAPPI is sharp and intense, which indicates it semicrystallinity nature. The diffraction pattern of Kapton shows amorphous features because of the broad, low intensity peaks, and no peaks indicative of crystalline phases. The crystallinity of the NAPPI can be explained based on the high degree of symmetry and regularity of molecular chain. The single-chain morphology containing ten repeat units of NAPPI and Kapton in Figure 7 was obtained by using Materials Studio molecular simulation. It can be seen that the chain of NAPPI have symmetrical and non-spiro linear rod-like chain structure, which has better regularity compared to the spiro chain structure of Kapton. Symmetry structure facilitates the regular arrangement of polymer chains to form a highly ordered lattice. In addition, the existence of rigid planar naphthalene structure improves the regularity of molecular chains, and the existence of polar groups enhances the interchain cohesion. The combined effect leads to regularity, planarity, and linearity of the polymer chain structure, which makes the NAPPI matrix have good crystallinity. Crystallization can significantly improve the barrier properties of polymers. This is because the crystalline phase is generally considered to be impenetrable even for small gas molecules, while permeating molecules can only penetrate through the amorphous phase [44,45]. Therefore, in the NAPPI system, the permeating molecules must bypass the crystallite area, which has a more tortuous diffusion path compared with amorphous Kapton. This is an important reason for the excellent barrier property of NAPPI.

The interplanar distance (*d*-spacing) at the maximum intensity in the WAXD spectrum is usually regarded as the inter-chain distance between polymer chains [46]. Table 3 summarizes *d*-spacing values and density values for Kapton and NAPPI films. It can be seen that NAPPI exhibits a smaller *d*-spacing than Kapton, which the *d*-spacing of NAPPI is 4.18 Å at *2θ* = 21.25°. The smaller *d*-spacing indicates the closer chain packing in NAPPI, which can be explained by its chain structure described above. Additionally, it can be found from Table 3 that the density of NAPPI (1.6142 g·cm^−3^) is larger than that of Kapton (1.4246 g·cm^−3^). In summary, the good crystallinity proves that the chains are arranged regularly and orderly, the lower interchain distance combined with higher density proves the tight chain packing, which is beneficial to enhancing the barrier properties of the polyimide films.

### 3.5. Free Volume Analysis

Free volume is one of the most important structural variables influencing permeation parameters in polymers. The free volume comprises the defects with atomic size and holes due to random stacking of atoms, permeate penetrates through the polymer matrix by using these defects and holes. Therefore, the size, number, distribution, and connectivity of the free volume determine the ability of the penetrant to penetrate through the polymer matrix. For the purpose of clearly explain the barrier mechanism of NAPPI at the molecular level. In this work, the free volume of polyimide films was measured by two different methods, experiment, and simulation. 

#### 3.5.1. PALS Method

**Figure 8:**
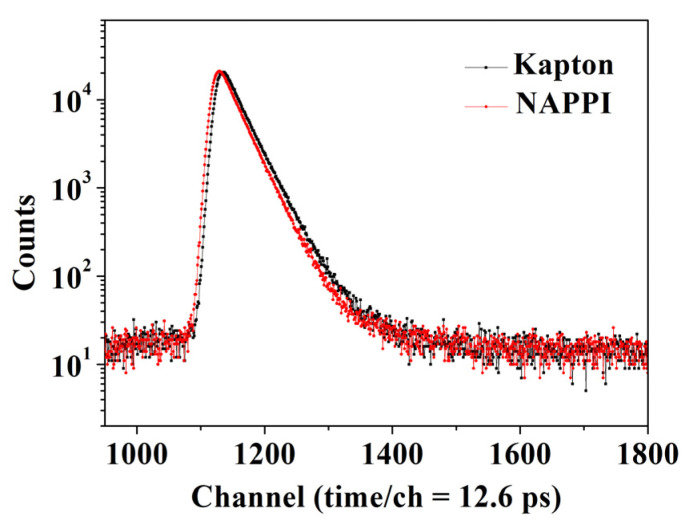
Positron lifetime spectra of Kapton and NAPPI.

Figure 8 shows the positron lifetime spectra of polyimide films. Table S2 summarizes the positron lifetime data collected for NAPPI and Kapton. It can be observed from the table that the composition of ortho-positronium (*o*-Ps) is extremely low. Therefore, the indefinite sphere model proposed by Tao et al. [47] cannot survey and evaluate the characteristics of free volume. Fortunately, Liao et al. [48] established a correction formula for calculating the mean free volume radius through the second component of the free positron lifetime (*τ*_2_), and this correction equation is particularly suitable for the polyimide system in this study. The correlation equation is as follows,

(1) $$\tau_{2}=0.260\times {\left[1-\frac{R}{R+3.823}+\frac{1}{2\pi }\sin\left(\frac{2\pi R}{R+3.823}\right)\right]}^{-1}$$

Assuming that the free volume cavity is spherical; Equations (2) and (3) can be used to obtain the fractional free volume (*FFV*).

(2) $$V_{f2}=\frac{4\pi R^{3}}{3}$$

(3) $$FFV=CI_{2}V_{f2}$$

where *C* is usually 0.0018 Å^−3^, *I*_2_ is the intensity of the second component, *V_f_*_2_ represents the size of the free volume [48,49,50].

Using the modified correlation equation, the data related to the free volume of NAPPI and Kapton are calculated and listed in Table 4. The NAPPI has a smaller mean free volume radius (2.2018 Å) and free volume size (44.68 Å^3^) than Kapton. More importantly, the corresponding relative *FFV* of NAPPI is much smaller than that of Kapton, where the *FFV* of NAPPI is 6.62% and that of Kapton is 11.55%. The smaller of the cavity and *FFV* can reduce the ability of penetrating molecules to penetrate through the polymer matrix, which is the fundamental reason why NAPPI has better barrier properties than Kapton.

#### 3.5.2. Molecular Simulations Method

The structure in the three-dimensional cubic unit cell of polyimides was constructed by molecular simulation, and the cell length is about 45 Å. Before the free volume analysis, the energy as a function of time and density as a function of time in simulation system are used to verify the equilibrium state. As shown in Figures S8 and S9, both the energy and density of the system have reached a stable state without major fluctuations, which prove that the system has reached equilibrium state. It is worth noting that the density of Kapton obtained by simulation is almost the same as the experimental density, this also proves that the simulation is reliable. However, the simulation density of the NAPPI system is only 1.53 g·cm^−3^, which is 4.96% lower than the experimental density. The smaller simulation density may be due to the existence of a large number of rigid groups and hydrogen bond interactions that make it difficult for amorphous cells to be further compressed. However, the error is less than 5% within the acceptable range. In addition, the *T_g_* of polyimide was simulated, Figure S10a,b show the volume as a function of temperature of simulated cell in NAPPI and Kapton, respectively. There is a distinct kink on each curve, indicating that the glass transition has occurred. The *T_g_* of NAPPI and Kapton are approximately 425 and 385 °C, respectively, which are very close to the experimental values. Because the density and glass transition temperature obtained by the simulation are consistent with the experimental data, the polymer model in this study can be considered reliable.

**Figure 9:**
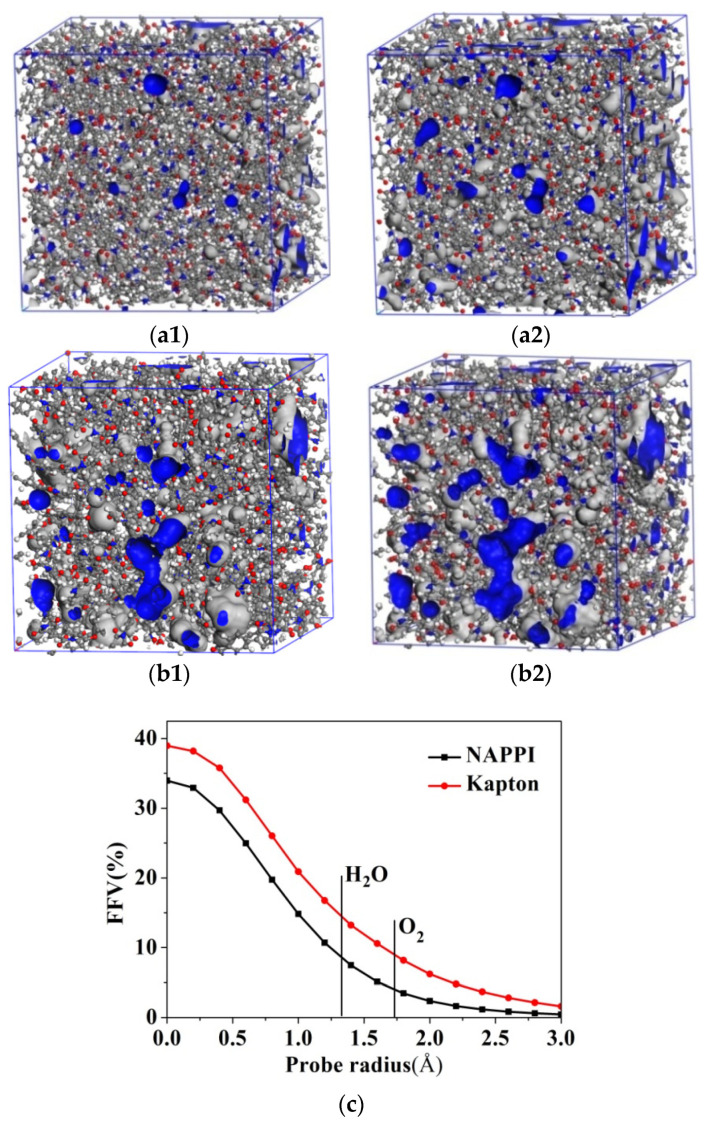
Morphologies of free volume in (**a1**,**a2**) NAPPI and (**b1**,**b2**) Kapton systems, the polymer networks are in gray, while the free volumes are denoted by blue with probe radius of 1.73 Å (**a1**,**b1**) and 1.325 Å (**a2**,**b2**), (**c**) *FFV* in NAPPI and Kapton as a function of probe radius.

This research is aimed at the barrier properties of oxygen and water vapor of NAPPI and Kapton, the purpose is to understand the available free volume that allows water and oxygen molecules to diffuse through the polymer matrix. Therefore, the kinetic radii of water and oxygen are selected as Connolly probes to estimate the free volume. The morphologies of free volume in NAPPI and Kapton systems are shown in Figure 9**.** The blue part in the figure represents the free volume of the polymer. It can be seen that the blue part in Figure 9(a1,a2) is significantly less than Figure 9(b1,b2). That means the O_2_ or H_2_O accessible free volume of the NAPPI is smaller than that of Kapton. In addition, almost no large voids appear in Figure 9(a1,a2), and there is no obvious interconnected between the voids. Therefore, it is difficult to form a continuous channel for penetrant molecules in NAPPI.

For quantitative explanation, Table 4 lists *FFV* calculated based on experimental and simulation methods. The *FFV* obtained from the simulation is smaller than the *FFV* estimated from PALS; this difference is due to the different size of the probe. Fortunately, the trend is consistent with the results estimated by PALS. The *FFV* of NAPPI which used the kinetic radius of water and oxygen as the probe is 8.58% and 3.98%, respectively.

The function relationship between *FFV* and probe radius in NAPPI and Kapton is shown in Figure 9c. *FFV* gradually decreases as the radius increases, and approaches zero when the radius is greater than 2.5 Å. In NAPPI system, the *FFV* with a radius of 1.0 Å is approximately 14.8%, which is significantly lower than that of Kapton and other common polyimide films (30–38%) [51,52]. In summary, the small free volume size and fraction confirms the tighter structure in NAPPI, which will restrict the penetration of permeate through the polymer matrix. It is precisely because of these morphological characteristics of free volume that NAPPI film has exceptionally excellent barrier properties.

### 3.6. Intermolecular Interaction Analysis

**Figure 10:**
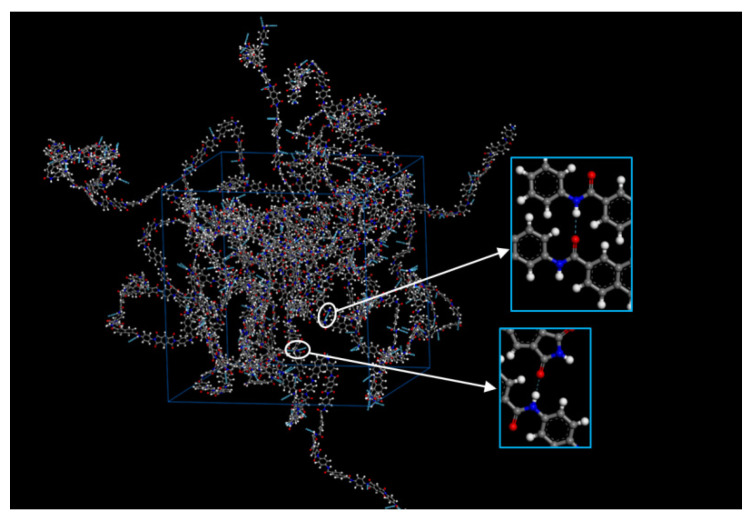
Hydrogen bonds in NAPPI amorphous cell (C, N, O and H are displayed in gray, blue, red, and white respectively, hydrogen bonds are shown as blue dotted lines).

The hydrogen bond interaction in NAPPI was analyzed by MD simulation. From Figure 10, it can be observed that two types of hydrogen bonds are formed. This is because the hydrogen on the amide group may form a hydrogen bond with the oxygen on the amide group and the oxygen on the imide ring. In this study, the hydrogen-acceptor distance less than 2.5 Å were registered as hydrogen bonds. It can be found from Table 4 that 139 hydrogen bonds are formed in NAPPI due to the introduction of amide groups. In addition, Table 4 also shows that NAPPI has a higher cohesive energy density (CED) of 629 J·cm^−3^, which may be caused by the existence of a large number of hydrogen bonds in the system.

**Figure 11:**
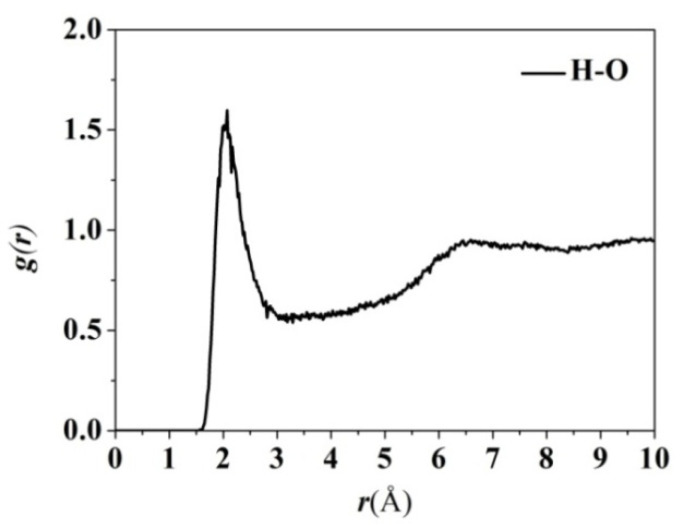
Radial distribution function (*g*_H-O_(*r*)) of H (in amide group) and O (in amide group and imide ring) in NAPPI.

The radial distribution function (*g*_H-O_(*r*)) of H (in amide group) and O (in amide group and imide ring) in NAPPI is used to further confirm the intermolecular interaction; the results are shown in Figure 11. The probability distribution of H and O is given by *g*_H-O_(*r*). A strong peak can be found around 1.8–3.0, and the distance between atoms of 2.6 and 3.1 corresponds to a hydrogen bond, which means that the interaction between the two atoms is likely to be a hydrogen bond [53]. Since the strongest physical force in the intermolecular interaction is the hydrogen bond, the existence of hydrogen bonds can increase the cohesion between chains and make chain packing more compact, thereby reducing the free volume of the polymer, and consolidating the formed crystalline conformation, which is very helpful to improve the barrier property.

### 3.7. Solution-Diffusion Analysis

Generally, the solution-diffusion mechanism is used to explain the permeability of gas in the polymer matrix. From the perspective of the permeation process, the barrier properties of polymers strongly depend on their sorption and diffusion behavior. A more intuitive expression is that the permeability (*P*) is the product of the solubility (*S*) and the diffusivity (*D*), which can be expressed by the Equation (4) [16].

(4) P=S×D

Permeability plays a decisive role in evaluating the barrier properties of polymers. Therefore, the barrier properties of the polymer can be improved by reducing the solubility of penetrant molecules in the polymer or limiting the diffusion of penetrant molecules in the matrix. In order to evaluate the permeability of H_2_O and O_2_ in NAPPI and Kapton, this study investigated the sorption and diffusion of gas molecules in the film.

#### 3.7.1. Sorption Analysis

**Figure 12:**
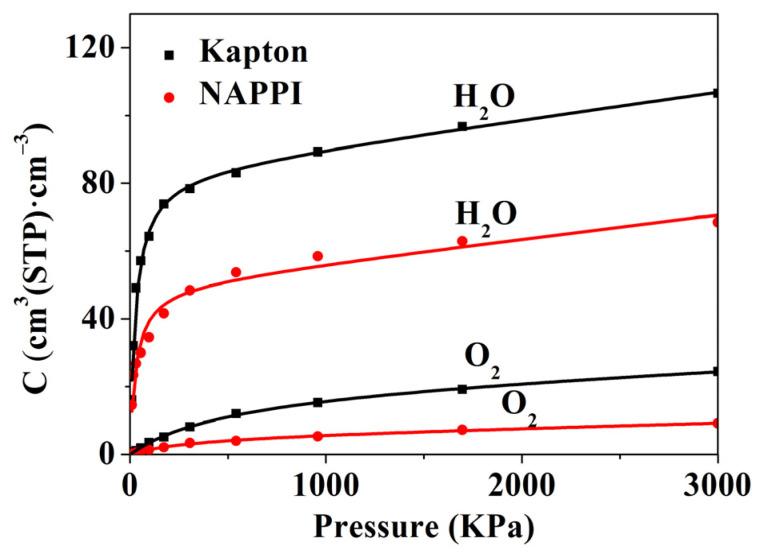
Sorption isotherms of O_2_ and H_2_O in NAPPI and Kapton.

The sorption of gas molecules in the polymer is obtained by GCMC simulation prediction. Figure 12 plots the sorption isotherms of H_2_O and O_2_ in Kapton and NAPPI, lower capacities of H_2_O and O_2_ in NAPPI was observed. This is a typical dual mode sorption isotherm. First, the total gas concentration (*C*) can be obtained through the dual-mode sorption model (Equation (5)), which means that there are two types sorption sites of Henry and Langmuir in the glassy polyimides. The solubility coefficient (*S*) can be calculated by Equation (6), which is the ratio of *C* to pressure [52,54,55].

(5) $$C=C_{D}+C_{H}=k_{D}p+\frac{C_{H}^{\prime }bp}{1+bp}$$

(6) $$S=\frac{C}{p}=k_{D}+\frac{C_{H}^{\prime }b}{1+bp}$$

This study uses the concept of infinite dilution to estimate the solubility, which means that the solubility is assumed when the gas concentration is very low, or the pressure is extrapolated to zero. Therefore, the solubility coefficient can be expressed by Equation (7):

(7) $$S=\underset{p\to 0}{lim}\frac{C}{p}=k_{D}+C_{H}^{\prime }b$$

**Figure 13:**
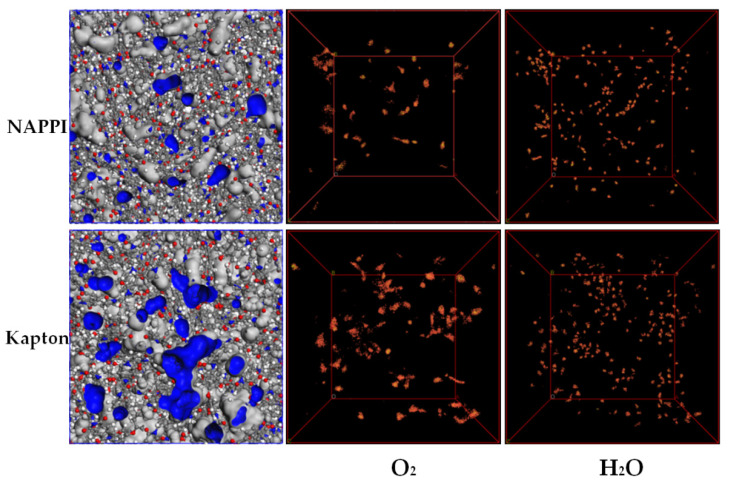
Sorption sites of O_2_ and H_2_O in NAPPI and Kapton.

Henry’s law coefficient *k_D_*, Langmuir hole affinity parameter *b* and the capacity parameter $C_{H}^{\prime }$ were obtained by fitting the sorption isotherm. Then the solubility coefficient was calculated by Equation (7) and summarized in Table 5. The solubility coefficient of H_2_O and O_2_ in NAPPI is lower than that of Kapton, the results accord with the sorption capacities. Figure 13 shows the sorption sites of O_2_ and H_2_O in NAPPI and Kapton. Obviously, the voids in the film are occupied by gas molecules. Compared with Kapton, the sorption loading of O_2_ and H_2_O in NAPPI is lower. This is consistent with the result that NAPPI has a smaller fraction free volume and voids size. In addition, it can be found that there are more sorption sites for H_2_O than O_2_ in NAPPI. This is mainly caused by two reasons. Firstly, the size of H_2_O is smaller than O_2_, and more importantly, because NAPPI contains a large amount of amides groups with strong affinity for H_2_O, it is easy to form hydrogen bonds with water molecules, which will facilitate the sorption of H_2_O. The amide group in NAPPI can interact with water molecules through strong hydrogen bonds, so that the water molecules may be “locked” in the film, which will greatly limit the diffusion of water molecules in the film. Moreover, the locked water molecules may occupy the voids in the polymer to further reduce the free volume, which helps to improve the barrier properties of the film [29,30,31].

**Table 5:** Solubility coefficients, diffusion coefficients and permeability coefficients for H_2_O and O_2_ in NAPPI and Kapton.

Polyimides	*S* ^a^		*D* ^b^		*P* ^c^	
	O_2_	H_2_O	O_2_	H_2_O	O_2_	H_2_O
NAPPI	0.018	1.70	5.2	2.2	0.09	3.74
Kapton ^d^	0.056	3.92	89.4	12.8	5.00	50.18

^a^ Units of (cm^3^ (STP)·cm^−3^·cm Hg^−1^); ^b^ (10^−8^·cm^2^·s^−1^); ^c^ (10^−8^ cm^3^ (STP)·cm·cm^−2^·s^−1^·cm Hg^−1^); ^d^ Data comes from Ref. [56].

#### 3.7.2. Diffusion Analysis

The diffusion of penetrating molecules in the polymer reflects the dynamic characteristics of the penetrating molecules and the polymer system, in order to understand this dynamics characteristic, MD simulation was used to obtain the MSD of the gas molecules in polymers. The diffusion coefficients can be estimated by using Einstein equation [57,58,59,60].

(8) $$D=\frac{1}{6N}\underset{t\to \infty }{lim}\frac{d}{dt}\overset{N}{\underset{i=1}{\sum }}{\langle \left|r_{i}\left(t\right)-r_{i}\left(0\right)\right|\rangle }^{2}$$

where *N* represents the number of diffused atoms in the system, *t* represents the diffusion time, *r_i_* represents the displacement at time *t*. ${\langle \left|r_{i}\left(t\right)-r_{i}\left(0\right)\right|\rangle }^{2}$ is the MSD of the atoms over time *t*.

**Figure 14:**
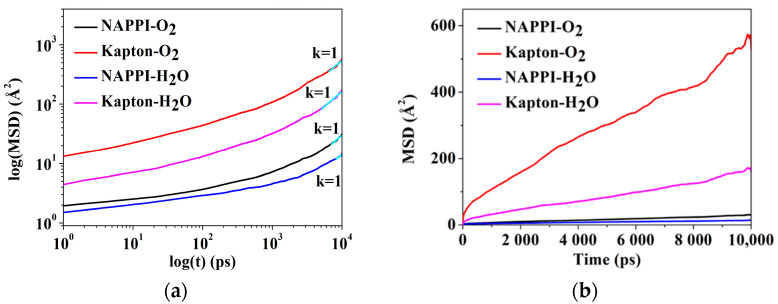
(**a**) log(MSD) and (**b**) mean square displacement (MSD) diagrams of H_2_O and O_2_ in polyimide as a function of time.

Figure 14a shows the MSD as a doubly logarithmic plot. By checking the slopes of the functions Log(MSD) and Log(t), it can be found that the slopes (k) of the four curves have all become one after a period of time, implying the occurrence of Einstein diffusion, which proves that Equation (8) is applicable. Figure 14b shows the MSD as a function of time for O_2_ and H_2_O in NAPPI and Kapton films, and the calculated diffusion coefficients are presented in Table 5. It can be seen that H_2_O and O_2_ have lower mobility in NAPPI film, and the diffusion coefficient of H_2_O and O_2_ in NAPPI is much lower than that of Kapton, which are 2.2 × 10^−8^·cm^2^·s^−1^ and 5.2 × 10^−8^·cm^2^·s^−1^, respectively. This indicates the diffusion of gas molecules in NAPPI is much more difficult than that in Kapton.

**Figure 15:**
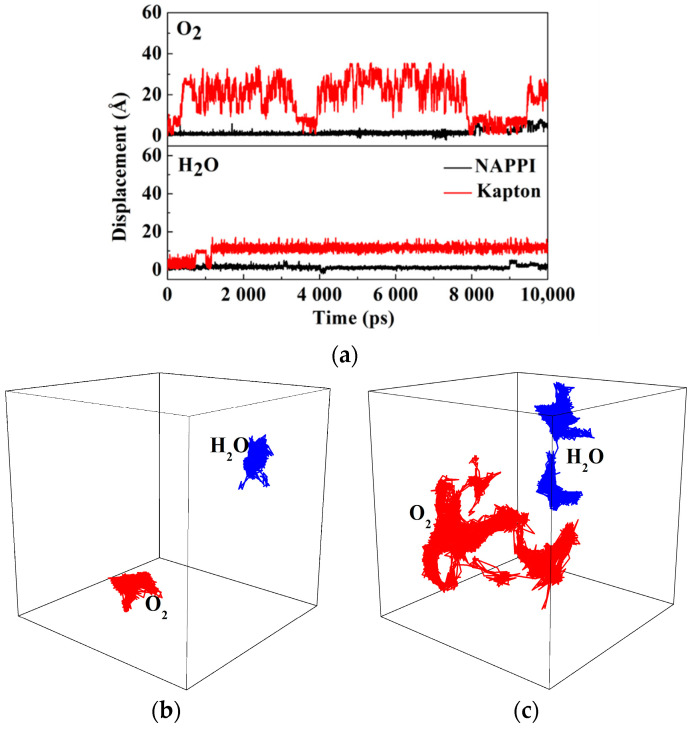
(**a**) Displacement and trajectories of H_2_O and O_2_ in (**b**) NAPPI and (**c**) Kapton.

To better understand the diffusion behavior, a detailed analysis was carried out by tracing the displacement and trajectory of H_2_O and O_2_ in the polymer matrix. The displacement of H_2_O and O_2_ in NAPPI and Kapton are shown in Figure 15a. The variation in displacement can be considered as a hopping mechanism. The significant variation of displacement means that the gas molecule may jump into adjacent voids, while steady vibration represents that gas molecules may be trapped in a void. It can be found that H_2_O and O_2_ jumped multiple times in Kapton, while in NAPPI they were almost trapped in their initial void. The diffusion trajectory of gas molecules in polyimide is shown in Figure 15b,c. Obviously, H_2_O and O_2_ exhibit low mobility in NAPPI. They are almost trapped and can only jump repeatedly in a small area. Nevertheless, the jumping motion becomes higher frequency and longer distance in Kapton. This is because the voids size in NAPPI is small and there is almost no interconnected between the voids which are discussed in free volume analysis. In addition, the strong chain rigidity of NAPPI is also an important reason.

In general, the process of penetrant molecules diffusing through the polymer film can be expressed as the possibility that gas molecules jump into a neighboring void through temporary channels formed by the continuous vibration of the chains [49]. Consequently, the mobility of polymer chains and chain packing can be considered as the key factors that direct affect diffusion. In NAPPI system, due to the presence of the naphthalene ring and the amide group increases the rigidity of the chain and leads to a strong inter-chain cohesion, so it is difficult to form a temporary channel for the diffusion of gas molecules. Therefore, gas molecule will have a high probability of being trapped in the void and unable to jump to the neighboring void, thereby restricting the diffusion.

After obtaining the solubility coefficient and diffusion coefficient, the permeability coefficient can be calculated by Equation (4). The calculation results are listed in Table 5. The permeability coefficients of O_2_ and H_2_O in NAPPI are much lower than Kapton. This is the fundamental reason why NAPPI has better barrier properties than Kapton. It is worth noting that the density of NAPPI in the simulation system is smaller than the real density, the permeability coefficient value may be smaller if the model reached the real density.

**Figure 16:**
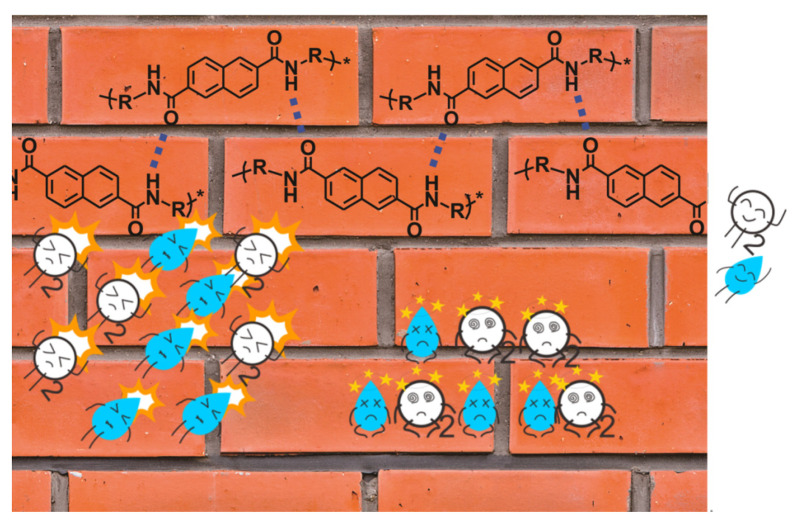
The barrier mechanism of NAPPI conceptually metaphorized as the “wall”.

The barrier mechanism of NAPPI can be conceptually metaphorized as the “wall” shown in Figure 16. The rigid naphthalene ring is assumed to be a “brick”, the strong hydrogen bond interaction formed between the amide groups can be regarded as “cement”. Driven by the strong intermolecular interaction formed by amide groups, polymer chains containing rigid naphthalene rings can be regularly arranged and tightly packed, just like stacking bricks into an orderly and sturdy wall under the bonding of cement. This is reflected in the smaller interplanar distance and free volume of NAPPI. The characteristic of NAPPI film to prevent gas from penetrating can be metaphorized as most gas molecules are blocked outside the “wall”, a small part of gas molecules are trapped in the “wall”, and only a few gas molecules can successfully pass through the “wall”. Although the actual arrangement of polymer chains cannot achieve this idealistic state, it is also helpful to understand the barrier mechanism of NAPPI.

## 4. Conclusions

A polyimide film (NAPPI) with excellent thermal stability and barrier properties obtained by polymerization of PMDA and NAPDA containing naphthalene ring and amide group was succeeded synthesized. Then, the relationship between barrier performances and structural characteristics of NAPPI was explored by combining experiment and simulation methods. The results revealed that the presence of naphthalene ring and amide group increased the chain rigidity and interchain cohesion, so that NAPPI have a small interchain distance and close chain packing, which leads to better crystallinity and smaller free volume size and fraction. These structural characteristics not only reduce the sorption sites of H_2_O and O_2_ on NAPPI, but also restrict their diffusion motion of in polymer matrix, resulting in poor permeability of NAPPI. In summary, experimental and simulation studies revealed that the introduction of naphthalene ring and amide group has a positive effect on the improvement of NAPPI barrier properties. This NAPPI film with excellent barrier properties and outstanding comprehensive performance is promising for application in high grade packaging area and flexible electronics encapsulation area, and its design concept has certain reference value for the development of novel high-barrier polymers.

## Figures and Tables

**Table 1:** Thermal and mechanical properties of the polyimide films.

Polymers	*T_g_* (°C)	*T_d5%_* (°C)	*T_d10%_* (°C)	CTE (ppm·K^−1^)	Tensile Strength (MPa)	Tensile Modulus (GPa)
Kapton ^a^	374	550	571	38.0	121 ± 3.0	2.3 ± 0.2
NAPPI	434	551	568	13.85	138 ± 3.0	3.5 ± 0.2

^a^ Data comes from Ref. [31].

**Table 2:** Barrier properties of Kapton and NAPPI.

Polyimide	Barrier Properties			
	OTR (cm^3^·m^−2^·day^−1^)	OP ^b^ (cm^3^·mil·m^−2^·day^−1^)	WVTR (g·m^−2^·day^−1^)	WVP ^c^ (g·mil·m^−2^·day^−1^)
Kapton ^a^	112.0 ± 0.80	330.7 ± 2.50	38.8 ± 0.72	114.5 ± 2.12
NAPPI	0.04 ± 0.002	0.13 ± 0.01	0.14 ± 0.01	0.42 ± 0.02

^a^ Data comes from Ref. [31]; ^b^ OP represents the oxygen permeation; ^c^ WVP represents the water vapor permeation.

**Table 3:** Physical properties of the NAPPI and Kapton.

Polymers	Density (g·cm^−3^)	*2θ* (°)	*d*-Spacing (Å)
Kapton ^a^	1.42	18.64	4.76
NAPPI	1.61	21.25	4.18

^a^ Data comes from Ref. [31].

**Table 4:** Simulated fractional free volume (*FFV*) and H-bonds numbers of polyimide films.

Polyimide	Density ^b^ (g·cm^−3^)	*R* (Å)	*V_f_*_2_ (Å^3^)	*FFV*^c^ (%)	*FFV*^b^ (O_2_, %)	*FFV*^b^ (H_2_O, %)	H-Bond (numbers)	CED (J·cm^−3^)
Kapton ^a^	1.41	2.6037	73.90	11.55	10.48	15.80	0	454
NAPPI	1.53	2.2018	44.68	6.62	3.98	8.58	139	629

^a^ Data comes from Ref. [31]; ^b^ data determined from molecular simulations; ^c^ data determined from experimentally.

## Data Availability

The data used to support the findings of this study are available from the corresponding author upon request.

## Supplementary Materials

The following are available online at https://www.mdpi.com/1996-1944/14/6/1402/s1, Figure S1: ^1^H NMR spectra of NAPDN. Figure S2: The MS spectra of NAPDN. Figure S3: The MS spectra of NAPDA. Figure S4: FT-IR spectrum of NAPDN and NAPDA. Figure S5: DSC and TG spectra of NAPDA. Figure S6: Photograph of NAPPI film. Figure S7: The stress-strain curves of polyimide. Figure S8: Energy as a function of time in simulation of (a) NAPPI and (b) Kapton. Figure S9: Density as a function of time in simulation of NAPPI and Kapton. Figure S10: Volume of simulation cell as a function of temperature in NAPPI and Kapton. Table S1: Thermal properties of NAPDA. Table S2: The positron lifetime data of Kapton and NAPPI.
