# Supplementary material for: Structure-Gas Barrier Property Relationship in a Novel Polyimide Containing Naphthalene and Amide Groups: Evaluation by Experiments and Simulations
Source: Materials (Basel). 2021 Mar 13;14(6):1402. doi: 10.3390/ma14061402 (PMC7999945; doi:10.3390/ma14061402)
Supplement: Supplementary file 1 [file materials-14-01402-s001.pdf]

materials

MDPI

Supplementary

Structure-Gas Barrier Property Relationship in a Novel Polyimide Containing Naphthalene and Amide Groups: Evaluation by Experiments and Simulations

Yi Zeng [1], Yiwu Liu [2,*], Jinghua Tan [2], Jie Huang [2], Junjie Liu [2], Ao Tang [2], Chengliang Chen [2] and Hong Chen [1,*]

[1] State Key Laboratory for Powder Metallurgy, Centre South University, Changsha, Hunan 410083, China; zengyi2426@163.com
[2] National and Local Joint Engineering Center of Advanced Packaging Materials R & D Technology, Key Laboratory of Advanced Packaging Materials and Technology of Hunan Province, School of Packaging and Materials Engineering, Hunan University of Technology, Zhuzhou 412007, China; tjh@hut.edu.cn (J.T.); huangjie3@sjtu.edu.cn (J.H.); junjie0108@whu.edu.cn (J.L.); tangao1234@163.com (A.T.); chenchengliangc@163.com (C.C.)
* Correspondence: liuyiwu@hut.edu.cn (Y.L.); chenhongcs@126.com (H.C.); Tel.: +86-0731-2218-2180(Y.L.)

This PDF file includes:

- Table S1–S2
- Figures S1–S10

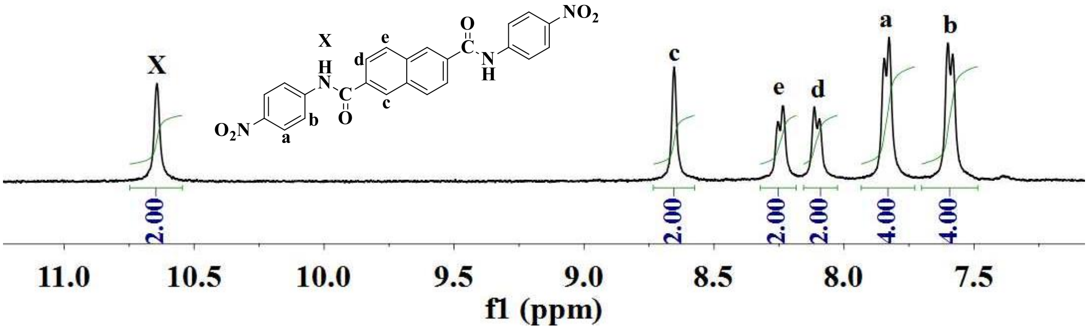

Figure S1. ^1H Chemical Shifts, f1 (ppm) in NAPDN ^1H NMR spectrum.

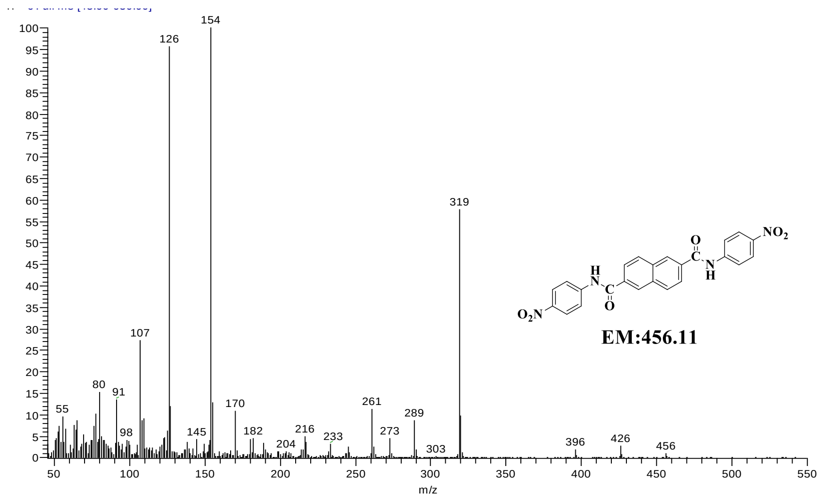

Figure S2. The mass charge ratio (m/z) of NAPDN molecular ion peak in MS spectra.

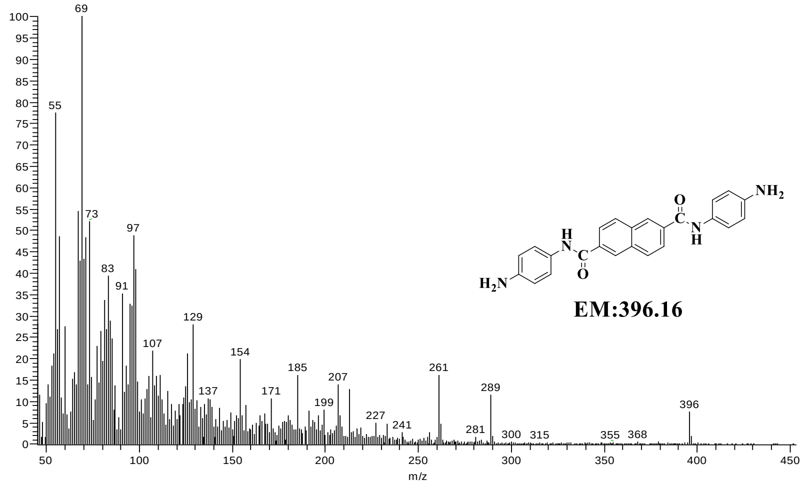

Figure S3. The mass charge ratio (m/z) of NAPDA molecular ion peak in The MS spectra.

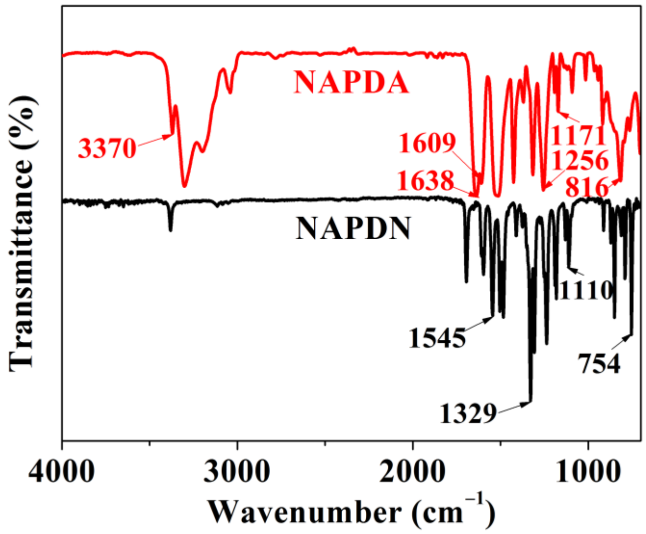

Figure S4. FT-IR spectrum of NAPDN and NAPDA.

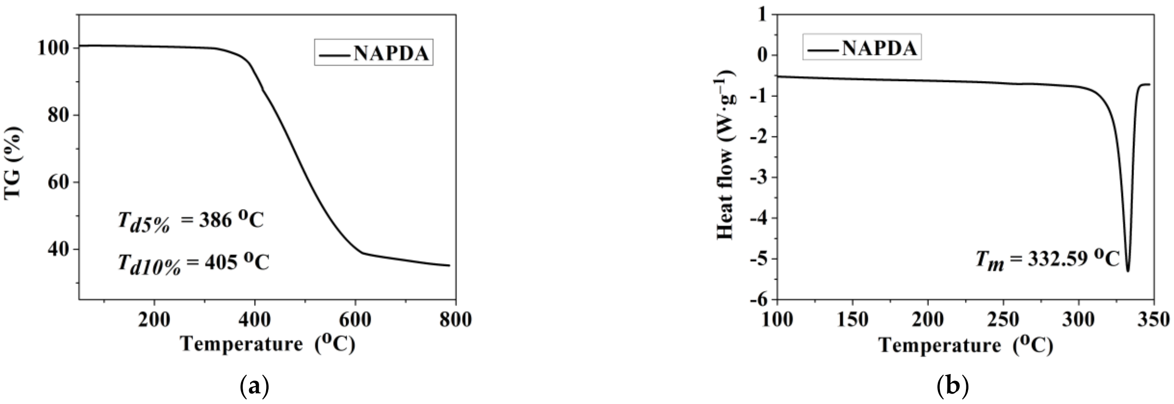

Figure S5. (**a**) TG and (**b**) DSC spectra of NAPDA.

Table S1. Thermal properties of NAPDA.

Compound	T_m (°C)	T_d (°C)	
		5 %	10 %
NAPDA	332.59	386	405

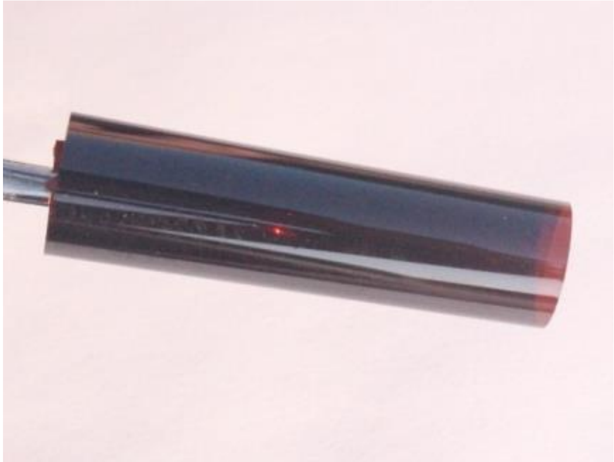

Figure S6. Photograph of NAPPI film.

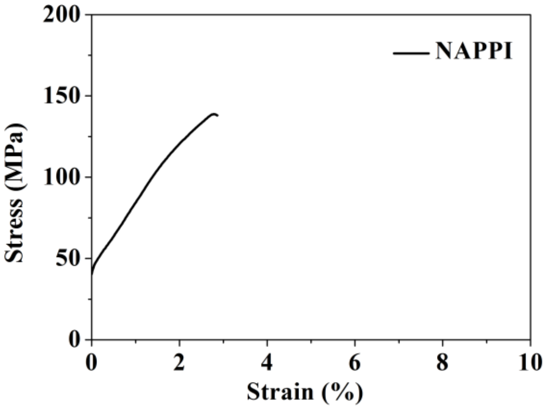

Figure S7. The stress-strain curves of polyimide.

Table S2. The positron lifetime data of Kapton and NAPPI.

Polyimide	τ_1(ns)	I_1(%)	τ_2(ns)	I_2(%)	τ_3(ns)	I_3(%)
Kapton	0.17	13.0	0.380	86.8	1.52	1.55
NAPPI	0.15	5.74	0.345	82.3	1.06	1.46

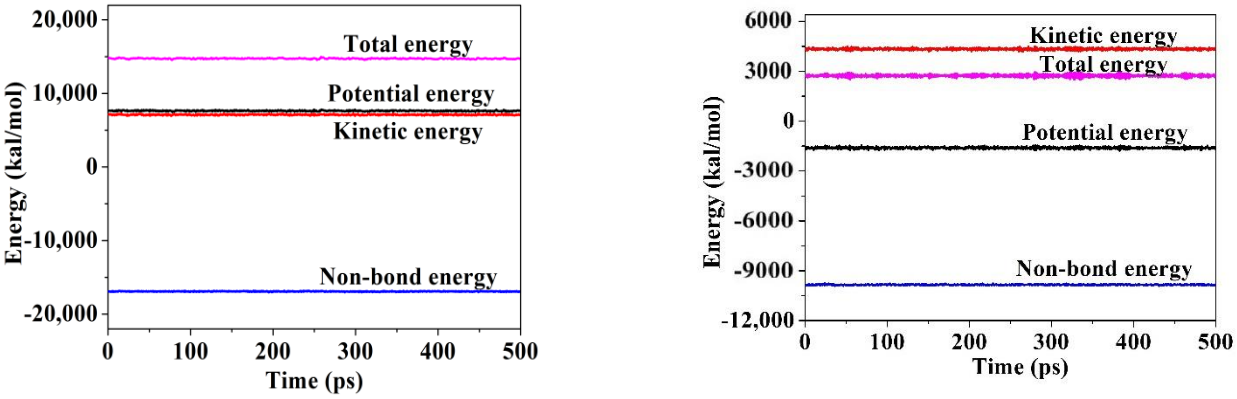

(a) (b)

Figure S8. Energy as a function of time in simulation of (**a**) NAPPI and (**b**) Kapton.

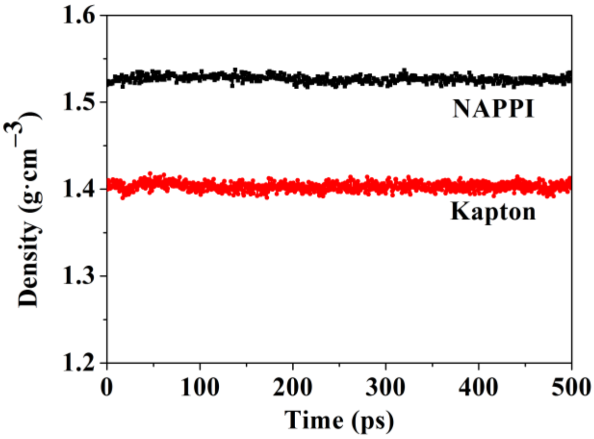

Figure S9. Density as a function of time in simulation of NAPPI and Kapton.

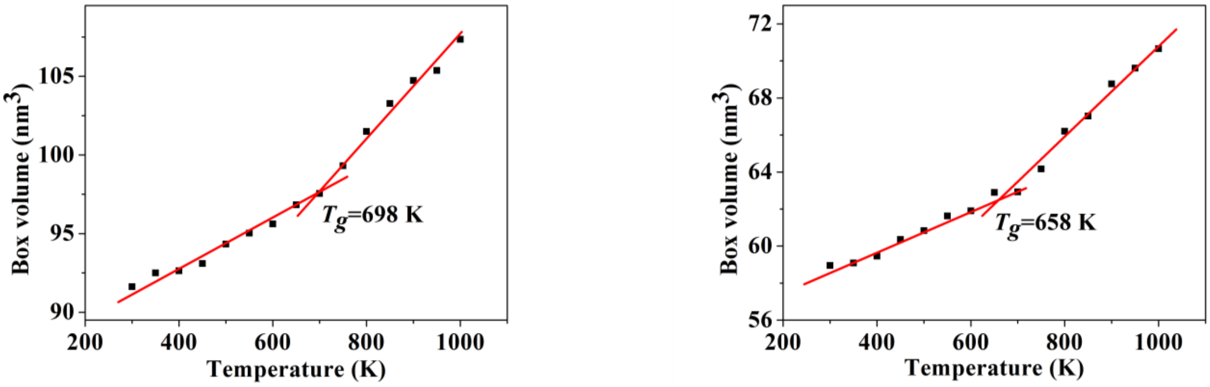

(a) (b)

Figure S10. Volume of simulation cell as a function of temperature in NAPPI (**a**) and Kapton (**b**).
